# Clinical Impact of New Reference Intervals for the Roche Prolactin II Immunoassay

**DOI:** 10.1210/jendso/bvae069

**Published:** 2024-04-09

**Authors:** Erin Earll, Bradley R Javorsky, Jenna Sarvaideo, Joely A Straseski, Robert D Nerenz

**Affiliations:** Medical College of Wisconsin, Milwaukee, WI 53226, USA; Department of Medicine, Division of Endocrinology and Molecular Medicine, Medical College of Wisconsin, Milwaukee, WI 53226, USA; Clement J. Zablocki VA Medical Center, Milwaukee, WI 53295, USA; Department of Medicine, Division of Endocrinology and Molecular Medicine, Medical College of Wisconsin, Milwaukee, WI 53226, USA; Clement J. Zablocki VA Medical Center, Milwaukee, WI 53295, USA; Department of Pathology, University of Utah Health Sciences Center, Salt Lake City, UT 84112, USA; Department of Pathology and Laboratory Medicine, Medical College of Wisconsin, Milwaukee, WI 53226, USA

**Keywords:** Prolactin, immunoassay, reference interval, bias, excess care

## Abstract

**Context:**

The Roche prolactin immunoassay is used throughout the world. It reports higher values than the Siemens immunoassay but the manufacturer-defined reference intervals are similar. Patient results are often above the Roche upper limit but within the Siemens interval, causing diagnostic confusion.

**Objective:**

Establish new reference intervals for the Roche and Siemens prolactin immunoassays.

**Methods:**

We established new reference intervals for the Roche and Siemens immunoassays using 374 specimens from healthy outpatients. We performed chart review for unnecessary testing and treatment for 298 patients in a 6-month period with at least 1 Roche prolactin value above the manufacturer-defined upper limit and below our new upper limit.

**Results:**

The new upper limit for the Roche assay was 37.8 ng/mL (females) and 22.8 ng/mL (males). The manufacturer-defined limits were 23.3 ng/mL and 15.2 ng/mL, respectively. New intervals for the Siemens assay matched the manufacturer. No cases of clinically significant pathophysiologic prolactin excess were identified in patients with values between the manufacturer-defined upper reference limit and our new Roche upper limit. Unnecessary further evaluation in these patients included 459 repeat prolactin measurements, 57 macroprolactin measurements, 39 magnetic resonance imaging studies, and 28 endocrine referrals. Eleven patients received dopamine agonists. The minimum cost of excess care using Medicare reimbursement rates was $34 134, with substantially higher amounts billed to patients and their insurance providers.

**Conclusion:**

Adoption of new upper reference limits for the Roche prolactin assay of 37.8 ng/mL (females) and 22.8 ng/mL (males) would not delay diagnosis or necessary intervention in patients with clinically significant pituitary tumors but would reduce unnecessary evaluation in patients without pathophysiologic prolactin excess.

Hyperprolactinemia is the most frequently encountered endocrine disorder caused by hypothalamic–pituitary axis dysfunction [1]. Prolactin, a 199 amino acid protein produced by lactotrophs of the anterior pituitary, promotes growth and development of mammary glands and milk production [2]. Its expression is under constant suppression by dopamine but plasma concentrations may increase in response to estrogens, thyrotropin-releasing hormone, nipple stimulation, stress, exercise, dopamine receptor antagonists, as well as other factors [3, 4]. Elevated plasma prolactin may also indicate the presence of a pathologic process, such as pituitary stalk compression or a prolactin-secreting pituitary adenoma [5]. Symptoms of prolactin excess in premenopausal females include amenorrhea or oligomenorrhea, infertility, galactorrhea, and decreased libido [6]. Males with prolactin excess rarely experience galactorrhea but generally endorse nonspecific symptoms of headache and decreased libido. In addition to the workup of these conditions, plasma prolactin may also be measured in the evaluation of transgender females initiating estrogen supplementation [7]. Values above the upper reference limit in a patient with suggestive signs and symptoms often require further clinical laboratory testing and/or imaging studies to determine the cause.

Prolactin is routinely measured in the clinical laboratory by 2-site, “sandwich” immunoassays performed on high-throughput, automated instruments but substantial bias exists between assays developed by different manufacturers [8-10]. Previous work comparing the Roche (Cobas) and Siemens (Centaur) prolactin assays found that values generated using the Roche assay were approximately 50% higher than corresponding Siemens values [11, 12]. Bias in automated immunoassays is a common phenomenon and can be mitigated by the establishment of assay-specific reference intervals. However, the upper limits of the manufacturer-defined Siemens reference intervals for males and females are higher than the corresponding manufacturer-defined Roche reference intervals. This is inconsistent with the high bias exhibited by the Roche assay and suggests that at least 1 reference interval needs to be adjusted. If the upper reference limit is too low, values in patients without prolactin excess will be incorrectly characterized as elevated, prompting unnecessary further evaluation. Conversely, if the upper reference limit is too high, cases of true prolactin excess may be missed, delaying diagnosis and appropriate medical intervention.

To improve interpretation of prolactin test results, we independently established new reference intervals for both the Siemens and Roche assays using the same set of remnant clinical specimens. To evaluate the effect of ethinyl estradiol on prolactin concentrations, we performed a separate reference interval calculation for females using ethinyl estradiol–containing contraceptives. To determine impact on care delivery, we performed chart review of patients with prolactin values above the upper limit of the manufacturer-defined reference interval and below the upper reference limit defined in this study to quantify unnecessary diagnostic testing and clinical intervention.

## Materials and Methods

### Collection and Processing of Remnant Clinical Specimens

Remnant serum or lithium-heparin plasma specimens collected at outpatient phlebotomy sites for clinical purposes from male or female patients aged 18-50 years were obtained from clinical laboratory refrigerated storage. Serum and lithium-heparin plasma are the specimen types recommended by both Siemens and Roche for prolactin measurement and are considered interchangeable for this purpose [13, 14]. Specimens were stored in primary tubes at 4 °C for less than 7 days, transferred to 2 aliquot tubes, and frozen at −20 °C. Specimens were in frozen storage for less than 7 days and thawed only once, immediately prior to prolactin measurement using the Roche and Siemens prolactin assays. Specimen handling, processing, and storage was performed in accordance with both manufacturers’ instructions. In order to identify samples collected for the routine screening of healthy individuals, test orders were limited to basic metabolic panel, comprehensive metabolic panel, 25-hydroxyvitamin D, hepatitis C antibody, human immunodeficiency virus antibody, or lipid panel. Chart review was performed to exclude samples from patients with any of the following conditions: pituitary adenoma, pregnancy, hypothyroidism, polycystic ovary syndrome, opioid use, antidepressant use/history of depression, menstrual irregularities, or placement of an intrauterine device. As the Siemens assay is not susceptible to biotin interference, biotin use was not included in the list of exclusion criteria. Consistent with current clinical practice and testing recommendations, female specimens were collected at any stage in the menstrual cycle. Although mean prolactin concentrations are lower in the follicular phase, interindividual variability is substantially greater than the change in mean concentration between different cycle phases [15, 16].

Specimens from males were evaluated as a single group (n = 127). As estrogen increases pituitary prolactin production, specimens from females were partitioned into 2 groups: those without any form of hormonal contraception (female, n = 125) and those prescribed and actively using combined ethinyl estradiol/progestin contraceptives (female EE, n = 122). A variety of hormonal contraceptives were used, including tablet (n = 104), vaginal ring (n = 15), and patch (n = 3). Daily ethinyl estradiol dose ranged from 10 to 35 µg. The full list of contraceptive preparations is listed elsewhere (Table S1 [17]).

### Immunoassay Methods

Prolactin was measured in all samples using the Elecsys Prolactin II assay on a Roche cobas e801 module (Indianapolis, IN. Analytical measurement range 0.09 to 470 ng/mL, coefficients of variation 2.7% at 28.1 ng/mL and 4.4% at 41.1 ng/mL) and the Siemens Prolactin assay on an Atellica IM instrument (Malvern, PA. Analytical measurement range 0.3-200 ng/mL, coefficients of variation 3.3% at 16.7 ng/mL and 2.3% at 37.2 ng/mL). Both are 2-site, sandwich immunoassays. The manufacturer-defined reference intervals for the Siemens assay were 2.8 to 29.2 ng/mL (female) and 2.1 to 17.7 ng/mL (male). The manufacturer-defined reference intervals for the Roche assay were 4.8 to 23.3 ng/mL (female) and 4.0 to 15.2 ng/mL (male). To rule out hypothyroidism and pregnancy, thyrotropin (TSH) (Elecsys TSH) and human chorionic gonadotropin (hCG) (Elecsys hCG + β) were measured on a Roche cobas e801 module in the 4 samples with the highest prolactin concentrations in each patient group. Similarly, macroprolactin testing was performed in these same samples following precipitation with polyethylene glycol 8000 solution (250 g/L, Sigma-Aldrich, St. Louis, MO). Monomeric prolactin (Siemens Atellica IM) less than 40% of the preprecipitation prolactin value was considered evidence of macroprolactin (cutoff established during internal validation study by the reference laboratory performing macroprolactin testing). TSH, hCG, and macroprolactin values ruled out hypothyroidism, pregnancy, and macroprolactin in all tested samples.

### Reference Interval Calculations and Statistical Analysis

Measured prolactin values were manually transcribed into Microsoft Excel and imported into R for statistical analysis and figure generation [18]. After Box–Cox transformation, outlier detection was performed by calculating the interquartile range and eliminating prolactin values outside the lower limit (lower quartile – (1.5 × interquartile range)) and upper limit (upper quartile + (1.5 × interquartile range)) [19, 20]. After removal of outliers and elimination of hypothyroidism, pregnancy, or macroprolactin as a cause of prolactin elevation in the samples with the 4 highest values in each group, reference intervals were calculated using the nonparametric method [21]. Significance of differences in prolactin values in patients prescribed ethinyl estradiol were calculated in Excel using a t-test (2-tailed, equal variance, *P* < .05 considered significant).

### Assessment of Clinical Impact of Revised Reference Intervals

Prolactin values generated using the Roche assay from January 1, 2022, to June 30, 2022, were obtained from the laboratory information system. Chart review was performed for patients with at least 1 value above the manufacturer-defined upper reference limit (female: 23.3 ng/mL, male 15.2 ng/mL) and below the upper reference limit established in this study (female: 37.8 ng/mL, male: 22.8 ng/mL). Authors with access to the medical record (E.E., B.R.J., J.S., R.D.N.) reviewed clinic notes, provider–patient correspondence, and clinical laboratory test results to determine whether subsequent clinical consultation, laboratory testing, imaging studies, and medical interventions were performed solely as a result of the prolactin value, solely due to patient presentation, or a combination of both. Cost estimates were calculated using published 2023 Medicare reimbursement rates for ICD codes 84146 (prolactin measurement, $19.38), 70553 (magnetic resonance imaging [MRI] sella, $337.52), A9585A (gadavist injection, $169.26), and a weighted average based on institutional use of 99203, 99204, 99205 (new patient visits following endocrine referral, $116.60).

### Human Subjects Protection Statement

This study was reviewed and approved by the Medical College of Wisconsin Institutional Review Board as a quality improvement initiative.

## Results

**Figure 1.**
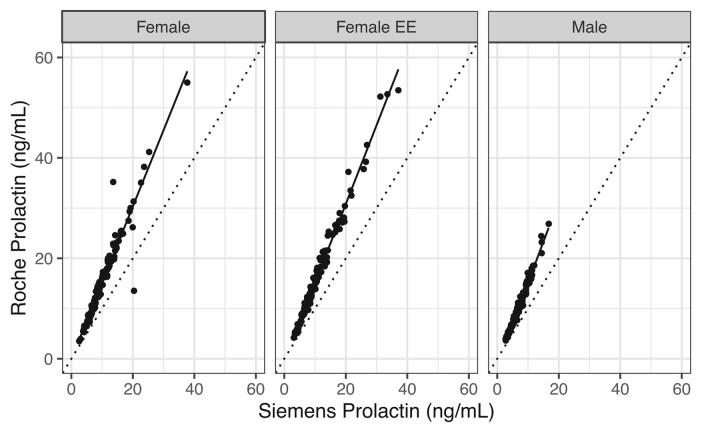
Comparison of concurrent prolactin values generated using the Siemens and Roche immunoassays on the same set of remnant clinical plasma or serum specimens. Female, female patients not prescribed ethinyl estradiol-containing contraceptives; Female EE, female patients prescribed ethinyl estradiol-containing contraceptives; Male, male patients. Linear regression equations: female: y = 1.52 × –0.098, R^2^ 0.923; female EE: y = 1.57 × –0.558, R^2^ = 0.984; male: y = 1.63 × –1.139, R^2^ = 0.976.

Mean ages and SDs for the female, female EE, and male groups were 30.4 (7.1), 29.1 (7.6), and 32.2 (7.2), respectively. As shown in Fig. 1, prolactin values measured by the Roche assay were approximately 55% higher than corresponding values generated by the Siemens assay, which is consistent with previous studies [11, 12]. Bias was consistent across all 3 patient subgroups (female: y = 1.52 × –0.098, R^2^ 0.923; female EE: y = 1.57 × –0.558, R^2^ = 0.984; male: y = 1.63 × –1.139, R^2^ = 0.976) as well as the full dataset of all study samples combined into a single cohort (all y = 1.56 × –0.561, R^2^ = 0.964).

Table 1 describes the female (2.8-29.2 ng/mL) and male (2.1-17.7 ng/mL) reference intervals defined by Siemens in their reference interval study. Reference intervals for the Siemens assay generated using remnant clinical specimens collected from healthy outpatients in southeast Wisconsin were 3.8 to 23.6 ng/mL (female, n = 125), 3.5-30.9 ng/mL (female EE, n = 122), and 2.9 to 14.5 ng/mL (male, n = 127).

**Table 1.** Siemens and Roche plasma prolactin reference intervals (ng/mL)

	Female		Female EE		Male	
	Low limit (90% CI)	High limit (90% CI)	Low limit (90% CI)	High limit (90% CI)	Low limit (90% CI)	High limit (90% CI)
Siemens (manufacturer defined) (n = 202 female, 139 male)	2.8	29.2	—	—	2.1	17.7
Siemens (this study) (n = 125 female, 122 female EE, 127 male)	3.8 (2.6-4.2)	23.6 (19.9-37.7)	3.5 (3.1-4.3)	30.9 (21.8-37.1)	2.9 (2.9-3.4)	14.5 (11.3-16.7)
Roche (manufacturer defined) (n = 198 female, 102 male)	4.8	23.3	—	—	4.0	15.2
Roche (this study) (n = 125 female, 122 female EE, 127 male)	5.3 (3.5-6.5)	37.8 (30-55)	5.0 (4.2-5.9)	51.5 (37.2-53.5)	4.2 (3.6-4.7)	22.8 (17.6-26.9)

Also shown in Table 1 are the manufacturer-defined female (4.8-23.3 ng/mL) and male (4.0-15.2 ng/mL) reference intervals for the Roche assay. Despite the previously documented high bias observed in the Roche assay relative to the Siemens assay, the manufacturer-defined upper reference limits for the Roche assay for both males and females are lower than those established by Siemens. Using the same set of remnant clinical specimens collected from healthy outpatients in southeast Wisconsin, newly established Roche reference intervals were 5.3 to 37.8 ng/mL (female, n = 125), 5.0 to 51.5 ng/mL (female EE, n = 122), and 4.2 to 22.8 ng/mL (male, n = 127). The manufacturer-defined upper reference limit for the Roche assay was outside the 90% confidence interval of the upper reference limit for both the female and male intervals established here.

From January 1, 2022, through June 30, 2022, 206 female patients and 92 male patients had at least 1 clinician-ordered plasma prolactin value between the manufacturer-defined Roche upper reference limit and the upper reference limit established in this study (female: 23.4-37.8, male: 15.3-22.8). Based on information in provider clinic notes, clinical laboratory test ordering patterns, and results of imaging studies, none of the 298 patients had a clinically significant, pathophysiologic cause of prolactin excess (Table 2). By contrast, substantial unnecessary further workup was performed in this patient group solely in response to prolactin values above the manufacturer-defined upper reference limit, including 459 repeat prolactin measurements, 57 macroprolactin measurements, 39 sella MRI studies, and 28 referrals to endocrine providers. Furthermore, 11 of these patients received dopamine agonist treatment despite unremarkable imaging studies and/or mild, nonspecific symptoms. Using published 2023 Medicare reimbursement rates for the relevant ICD codes, this unnecessary testing collectively totaled $34 134 for this 6-month period. However, as Medicare reimbursement was used for these calculations, this figure almost certainly underestimates the amount billed to patients and their insurance providers.

**Table 2.** Retrospective chart review summary and assessment of impact of new Roche reference intervals (January 1, 2022, to June 30, 2022)

	Female	Male
Prolactin values (Roche)	23.4-37.8 ng/mL	15.3-22.8 ng/mL
Total number of patients	206	92
Patients with clinically significant prolactin excess whose care would have been adversely affected by a higher upper reference limit	0	0
Patients with a Roche prolactin value between the manufacturer- and study-defined upper reference limits with follow-up testing prompted by a prolactin value only	93	33
Unnecessary prolactin measurements solely due to prolactin value	241	218
Unnecessary macroprolactin measurements solely due to prolactin value	49	8
Unnecessary sella magnetic resonance imaging scans solely due to prolactin value	20	19
Unnecessary endocrine referrals solely due to prolactin value	25	3
Patients receiving dopamine agonists solely due to prolactin value	9	2

**Figure 2.**
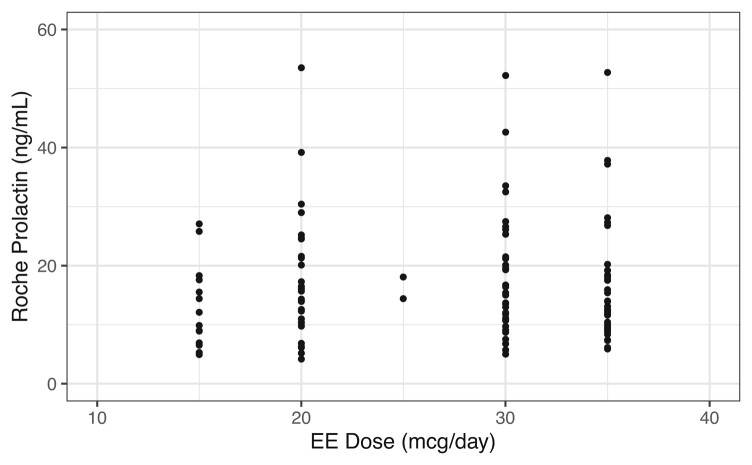
Roche prolactin values classified by daily ethinyl estradiol dose in the group prescribed ethinyl estradiol-containing contraceptives. t-test *P* values 15 vs 20 µg/day: 0.212, 15 vs 30 µg/day: 0.149, 15 vs 35 µg/day: 0.321, 20 vs 30 µg/day: 0.794, 20 vs 35 µg/day: 0.717, 30 vs 35 µg/day: 0.525.

As shown in Fig. 2, there was no dose–response relationship observed between ethinyl estradiol and plasma prolactin concentrations measured using the Roche assay. Prolactin values did not increase with increasing ethinyl estradiol dose and were not significantly different in patients receiving 15, 20, 25, 30, or 35 µg/day ethinyl estradiol.

## Discussion

We chose to undertake this study after encountering numerous patients with discrepant plasma prolactin values measured using the Roche and Siemens immunoassays. In these cases, values generated using the Roche assay were above the manufacturer-defined upper reference limit; however, when the same specimens were sent to a reference laboratory for testing using the Siemens assay, values were well within the Siemens reference interval. This discrepancy caused substantial confusion for the affected patients and their care providers as 1 result suggested the need for continued evaluation of prolactin excess while the other excluded prolactin excess from further consideration. Previous work and unpublished observations from other clinical laboratories serving geographically distinct patient populations collectively support raising the upper reference limit of the Roche assay [22]. Reference intervals for the Siemens assay generated in this study using remnant clinical specimens are consistent with those developed by the manufacturer, supporting use of the manufacturer-defined interval to guide interpretation of values generated using the Siemens assay. This finding also supports the validity of our approach to establish new reference intervals using selected remnant clinical specimens.

Our study also demonstrates that the Roche-defined upper reference limits should be increased, consistent with the known bias between the Roche and Siemens assays. It is unclear why the 2 manufacturer-defined reference intervals did not account for this bias. One explanation may be that during the manufacturers’ initial reference interval studies, the assays generated concordant results but gradually drifted apart due to small, incremental changes in calibrator preparations or other assay components. As the measured bias in our study is similar to previous reports published in 2019, substantial changes in assay performance over the intervening years seem unlikely, but it is possible that assay drift or shift occurred prior to that point [11]. Another possible explanation is that bias between the 2 assays has remained constant but the 2 manufacturers used different inclusion and exclusion criteria when selecting reference individuals, resulting in reference populations with very different characteristics. It is difficult to determine whether this is a contributing factor as neither manufacturer defines inclusion/exclusion criteria in their package inserts, instead describing their reference populations simply as “apparently healthy individuals.”

Although biotin use was not used as an exclusion criterion, it is unlikely that biotin interference substantially affected the findings presented here. The Siemens assay is not susceptible to biotin interference as the capture antibody is covalently bonded to the magnetic microparticles. The fact that the Roche-Siemens bias was similar across male and female subgroups (high-dose biotin use is more prevalent in females) and consistent with previously published work indicate that biotin interference is not a substantial concern.

We also show that adoption of higher reference limits for the Roche assay is unlikely to adversely impact care delivery by delaying diagnosis or preventing initiation of medically necessary treatment in patients with true prolactin excess. In our 6-month retrospective review, patients with plasma prolactin values above the manufacturer-defined upper reference limit but below the new upper reference limit defined here fell into 1 of 2 categories. The first consisted of patients with a confirmed, previously diagnosed cause of prolactin excess whose plasma prolactin values at initial presentation were >100 ng/mL, exhibited classic signs and symptoms, and underwent imaging studies that identified a pituitary tumor. Their symptoms had resolved following treatment with dopamine agonists or surgical intervention and they were undergoing routine monitoring at regular intervals. For these patients, follow-up prolactin measurements were performed regardless of the previous prolactin value and were not considered unnecessary. The second category consisted of patients with mild, nonspecific symptoms whose plasma prolactin values either remained just above the manufacturer-defined upper reference limit or decreased to within reference limits spontaneously without medical intervention. If performed, MRI of the sella did not identify any abnormalities and concurrent plasma prolactin measurement using an alternate test method generated values within that assay-specific reference interval. These patients were considered not to have a pathophysiologic cause of prolactin excess, did not need further clinical laboratory testing or imaging studies and would not have had necessary intervention delayed or withheld if their initial plasma prolactin value had been characterized as “normal.” They would, however, have been spared the cost, anxiety, and potential adverse events associated with unnecessary further workup and treatment. It is theoretically possible that a patient with pathologic hyperprolactinemia could present with a value below the new upper reference limits presented in this study. However, this is very unlikely given the new limits were derived from a robust sampling of reference individuals and verified by a 6-month retrospective review of 298 patients with prolactin values just below the new upper reference limits.

Data presented here also highlight the importance of clinical laboratories verifying manufacturer-defined reference intervals, which can be performed using 20 samples from a local reference population [23]. If 90% (18/20) of values fall within the manufacturer-defined limits, the interval has been verified as appropriate for the local patient population and can be adopted for clinical use. However, clinical laboratories may not always verify manufacturer-defined reference intervals for each assay they perform. Furthermore, although practice guidelines indicate that 20 specimens are sufficient, testing of additional specimens may be required for a robust evaluation of the manufacturer-defined interval. In our study, 18 of the first 20 sequential female (no EE) specimens and all of the first 20 sequential male specimens fell within the Roche-defined intervals. While these initial data would have been sufficient to adopt the manufacturer-defined intervals, collection of subsequent specimens showed that the Roche-defined upper limits were not appropriate for our patient population. Adoption of inaccurate reference limits can have profound negative consequences, a point highlighted by the many unnecessary MRIs and additional laboratory tests performed solely because of a falsely “elevated” prolactin value. While 18/20 within the reference interval is considered evidence of verification by consensus guidelines, inclusion of additional specimens may be required for a more robust evaluation.

It is important to note that our study provides limited clarity on the effect of EE-containing contraceptives on measured prolactin values. As the upper reference limit was higher in the subgroup of women using EE-containing contraceptives, it is reasonable to conclude that these contraceptive preparations increase circulating prolactin and require the application of a higher reference limit. However, this increase in the upper reference limit can be attributed to 2 additional samples with values >50 ng/mL in the EE group relative to the no EE group. These samples were not identified as outliers by the statistical test described earlier and were retained in the reference interval calculation. Visual inspection of the full spread of individual data points in Fig. 1 did not reveal consistently higher values in the EE group relative to the no EE group, instead showing a very similar distribution in the 2 groups. Furthermore, higher doses of EE did not correlate with higher prolactin concentrations. In clinical practice, the no EE reference interval will be applied to all values from female patients, regardless of contraceptive use. A comment indicating that estrogen supplementation may increase prolactin values will be included as an interpretive comment with each reported result.

Our study has limitations, primarily the relatively small sample size. Inclusion of additional data points would make the reference limits more robust and tighten the 90% CIs. However, our study included a larger number of male participants than the original Roche reference interval study and contains at least 120 samples in each subgroup, which is sufficient to establish a valid reference interval using the nonparametric method [23]. Our study also did not define reference intervals for postmenopausal women. These were established by Siemens in their original reference interval study and can be estimated for the Roche assay using the Roche-Siemens correlation equation presented here.

In summary, this work demonstrates the importance of verifying manufacturer-defined reference intervals and supports the adoption of higher reference limits for the Roche prolactin II assay, which is commonly used in clinical laboratories throughout the world. Use of the new reference limits established here will reduce unnecessary follow-up testing and treatment while continuing to identify patients with clinically significant, pathophysiologic prolactin excess.

## Disclosures

E.E., J.S., and J.A.S. have nothing to declare. B.R.J. has received research funding from Amryt Pharma. R.D.N. has received lecture fees and research funding from Abbott Diagnostics.

## Data Availability

Restrictions apply to the availability of some or all data generated or analyzed during this study to preserve patient confidentiality or because they were used under license. The corresponding author will on request detail the restrictions and any conditions under which access to some data may be provided.

## Abbreviations

EE: ethinyl estradiol/progestin
hCG: human chorionic gonadotropin
MRI: magnetic resonance imaging
TSH: thyrotropin
